# *In vivo* and *In vitro* Drug Interactions Study of Glimepride with Atorvastatin and Rosuvastatin

**DOI:** 10.4103/0975-1483.63169

**Published:** 2010

**Authors:** VJ Galani, M Vyas

**Affiliations:** 1 *Department of Pharmacology, A. R. College of Pharmacy and G. H. Patel Institute of Pharmacy, Vallabh Vidyanagar – 388120, Gujarat, India*

**Keywords:** Atorvastatin, drug interaction, glimepride, rosuvastatin

## Abstract

Aim of this investigation was to study the *in vivo* and *in vitro* drug interaction of glimepride with atorvastatin and rosuvastatin. *In vitro* drug interaction of glimepride with atorvastatin and rosuvastatin was studied using human pooled liver microsomes and evaluated using high performance liquid chromatography. *In vivo* pharmacokinetic drug interaction of glimepride (6 mg/kg) in coadministration with atorvastatin (60 mg/kg) and rosuvastatin (60 mg/kg) were studied in rats and analyzed using liquid chromatography tandem mass spectrometry (LC–MS/MS). In *in vitro* study, atorvastatin decreased its own metabolism as well as the metabolism of glimepiride. Rosuvastatin coadministration with glimepride reduced the metabolism of glimepride and increased the metabolism of its own. In *in vivo* study, concentration in plasma, C_max_, AUC_(0–t)_ and AUC_(0–∞)_ (area under the concentration-time curve, AUC) of glimepride was increased significantly in coadministration with atorvastatin whereas there was no significant change was observed in the case of coadministration with rosuvastatin. Half life (T_1/2_) and volume of distribution (V_d_) of glimepride decreased significantly with both atorvastatin and rosuvastatin. Elimination rate constant, K_el_ of glimepride increased significantly with both atorvastatin and rosuvastatin. Clearance (Cl) of glimepride decreased significantly but the decrease was more with atorvastatin than with rosuvastatin. It is concluded that glimepride metabolism is little affected by rosuvastatin *in vitro*, which agreed with the negligible interaction in *in vivo* study. Thus, from safety point of view rosuvastatin is better to prescribe as a coadministration therapy with glimepiride. On the other hand, atorvastatin could cause an increase in the bioavailability of glimepride per oral and also significantly decrease the metabolism of glimerpride in *in vitro* study. This may pose a positive implication in clinical practice.

## INTRODUCTION

Diabetes mellitus is a very commonly occurring metabolic disorder characterized by hyperglycemia and altered metabolism of lipids, proteins, and carbohydrates and occurs due to absolute or relative deficiency of insulin or insulin resistance.[1] Diabetes mellitus is associated with oxidative stress induced micro- and macrovascular complications. Long-term complications of diabetes mellitus involve almost all the vital organs such as heart, eyes, kidney, blood vessels, and nervous system. These complications lead to the development of obesity, hypertension, dyslipidemia, and insulin resistance.[2] There is a close association between complications of diabetes and diabetic dyslipidemia. Diabetic dyslipidemia accounts for around 80% diabetic deaths due to cardiovascular complications. There is a growing body of evidence to show that hyperglycemia and dyslipidemia are associated with an excessive cardiovascular risk.[3] Lipid lowering drugs (statins) are prescribed in patients of diabetes for the prevention of complications of diabetes like cardiovascular diseases or diabetic dyslipidemia.[4,5] The greatest effects are seen with the most potent statins such as simvastatin, atorvastatin, and rosuvastatin in higher doses.[6] One study showed that newer statin rosuvastatin and established statin atorvastatin have similar efficacy in reducing low density lipoprotein (LDL) in patients with diabetic dyslipidemia.[7] Glimepiride is a widely used third-generation sulfonylurea suitable for once daily administration in treatment of type 2 diabetes mellitus. It is completely absorbed after oral administration and is eliminated mainly via metabolism by cytochrome P450 (CYP) 2C9. The oral bioavailability of glimepiride is close to 100%.[8] Atorvastatin is metabolized by CYP3A4 isoenzyme.[9] Rosuvastatin is not extensively metabolized, but has some interaction with the CYP2C9 enzyme.[10] Many adverse drug—drug interactions of clinical interest can be attributed to the pharmacokinetic and pharmacodynamic changes that occur due to the alterations in hepatic drug metabolic pathways catalyzed by the CYP system. Statins are mainly considered for long-term use and often constitute part of a multiple-drug regime, which commonly leads to drug interactions. As statins do not differ in their pharmacodynamic properties, the difference in their pharmacokinetic profiles constitutes the rationale for choosing a specific statin suitable for combination therapy.[11]

Drug–drug interaction has become one of the major concerns not only for physician during the treatment of patients but also for pharmaceutical industries during the development of new drugs.[12] Glimepride and atorvastatin are metabolized by the different metabolic pathways whereas glimepride and rosuvastatin are metabolized by the same metabolic pathway. As cardiovascular problems are more common in diabetics, the possibility for the simultaneous use of such combination is more. *In vitro* study has become a critical first step in the assessment of drug interactions. Well-executed *in vitro* studies can be used as a screening tool for further *in vivo* assessment and can provide the basis for the design of subsequent *in vivo* drug interaction studies.[13] Hence, this study was designed to assess the *in vitro* drug interaction of glimepride–atorvastatin and glimepride-rosuvastatin and correlate it with single dose pharmacokinetic drug interaction of glimepiride in coadministration with atorvastatin and rosuvastatin in Wistar rats.

## MATERIALS AND METHODS

### Drugs and chemicals

Glimepride, Atorvastatin and Rosuvastatin (working standard) were obtained as gift samples from Cadila Healthcare Ltd., Ahmedabad, Gujarat. Trifluoro acetic acid (Sigma Aldrich), acetonitrile (Zydus Cadila), methanol (Merck), reduced nicotinamide adenine dinucleotide phosphate (NADPH) (Sigma Aldrich), monobasic potassium hydrogen phosphate (Merck), magnesium chloride (Qualigens), potassium hydroxide pellets (Merck) and human pooled liver microsomes (BD Gentest, USA) were used for the *in vitro* study. Carboxymethyl cellulose (Sigma), Tween-80 (Merck) and Milli-Q water were used for drug solutions preparation for the *in vivo* study.

### *In vitro* drug interaction study

#### Methodology

Glimepiride, atorvastatin and rosuvastatin were mixed with diluent [methanol : acetonitrile : water (40 : 40 : 20)] to a final stock solution of concentration 1 mg/ml. Glimepride + atorvastatin were diluted with the diluent to get a final stock solution of concentration 500µg/ml for both the drugs. Glimepride + rosuvastatin were also diluted with the diluent to get a final stock solution of concentration 500µg/ml for both drugs. Then, 5µl of glimepiride, atorvastatin, rosuvastatin, glimepiride + atorvastatin and glimepiride + rosuvastatin stock solutions were incubated with 215µl of diluted human pooled liver microsomes (final concentration 1.0 mg/ml) for 15 min at 37°C at 80 rpm in a shaking water bath and 100 µl of each of the five aliquots is transferred into 2 ml micro—centrifuge tubes. Then, 25 µl of preincubated NADPH (21.13 mM) solution was added and the solutions were incubated at 37°C and 80 rpm for 30 min in a shaking water bath. To stop the reaction, 200 µl acetonitrile was added in the first, second, third, fourth and fifth aliquots at 0, 15, 30, 45 and 60 min of incubation respectively and they were mixed for 1 min. The samples were centrifuged at 10,000 rpm for 5 min. Supernatants were assayed for the presence of substrate using a validated, sensitive, and specific isocratic high-performance liquid chromatography (HPLC) method. The supernatants were mixed with buffer and directly injected.

#### HPLC analysis

HPLC apparatus consisting of Kromasil C18 250 × 4.6 mm, 5 µ column was used for the analysis. Biphasic mobile system (A : B), reservoir A (0.1% trifluoroacetic acid in water) and reservoir B (0.1% trifluoroacetic acid in acetonitrile) was run as per the gradient program (time (min)/%B conc. v/v: 0.01/25.0, 3.00/40.0, 5.00/50, 7.00/70.0, 10.0/90.0, 12.0/90.0, 14.0/25.0, 18.0/25.0) with a total flow rate of 1 ml/min through the column to elute the analytes and elutes were monitored by the Agilent HPLC-UV with chemstation software at 237 nm. Percentage metabolism of drugs was calculated using the following formula.

$$\%\;\mathrm{metabolism}\;\mathrm{at}\;60\;\min\;=\;1\;-\;\frac{\mathrm{Area}\;\mathrm{of}\;\mathrm{analyte}\;\mathrm{test}\;\mathrm{at}\;60\;\min}{\mathrm{Area}\;\mathrm{of}\;\mathrm{analyte}\;\mathrm{test}\;\mathrm{at}\;0\;\min}\;\times \;100$$

### In vivo drug interaction study

#### Animals

Wister rats (200-250 g) of either sex bred in Central Animal House facility of the Zydus Research Centre, Ahmedabad, were used. The animals were housed under standard conditions, maintained on a 12-h light/dark cycle and had free access to food and water up to the time of experimentation. The animals were acclimatized to the laboratory environment 1 h before the experiments. Experiment was conducted during the light period (08:00-16:00 h). Experimental protocol was approved by the Institutional Animal Ethical Committee. Experiments were conducted according to the guidelines of Committee for the Purpose of Control and Supervision of Experiment on Animals (CPCSEA).

#### Preparation of drugs

Glimepiride, rosuvastatin, and atorvastatin solutions were formulated in 5% Tween-80 and 0.5% carboxy methyl cellulose in Milli-Q water for *in vivo* study.

#### Methodology

Freshly prepared solutions of glimepiride, atorvastatin, rosuvastatin, glimepiride + atorvastatin and glimepiride + rosuvastatin were administered as single oral dose to five groups of rats (n = 6). The selection of dose levels was based on efficacy dose and toxicokinetic doses.[14] Group I received glimepride (6 mg/kg, p.o.), group II received atorvastatin (60 mg/kg, p.o), group III received rosuvastatin (60 mg/kg, p.o.), group IV received glimepiride (6 mg/kg, p.o.) + atorvastatin (60 mg/kg, p.o), and group V received glimepiride (6 mg/kg, p.o.) + rosuvastatin (60 mg/kg, p.o.). Serial blood samples in heparinized saline solution were collected from retro-orbital plexus, approximately 0.3 ml at each time point namely 0 min (pre-dose), 10 min, 20 min, 40 min, 1 hr, 2 hr, 4 hr, 6 hr, 8 hr and 24 hr postoral dosing. Blood samples were centrifuged at 6000 rpm for 6 min to obtain the plasma. Methanol was added as a protein precipitating agent to plasma samples and vortexed for 1 min and centrifuged at 10,000 rpm for 5 min. The supernatant was transferred to liquid chromatography tandem mass spectrometry (LC–MS/MS) vial and analyzed. The concentrations of glimepiride, rosuvastatin, and atorvastatin were determined in each plasma sample using a validated LC-MS/MS method.

#### LC-MS/MS analysis

Plasma concentrations of drug metabolites were quantified by LC-MS/MS [WATERS ALLIANCE System (2695) connected to WATERS Micromass Quattro Micro triple quadrupole LC-MS/MS]. A Phenomenex Gemini C18 column (50 × 4.6 mm, 5 µm) and a mobile phase consisting of 0.1 % ammonia in HPLC grade water and methanol were used. Elutes were monitored by the analyst software. The ion transitions monitored were 489.22 m/z to 225.07 m/z for glimepride, 557.08 m/z to 397.00 m/z for atorvastatin and 489.22 m/z to to 86.68 m/z for rosuvastatin.

#### Analysis of pharmacokinetic parameters

Pharmacokinetic parameters of glimepiride, rosuvastatin, atorvastatin, glimepiride + rosuvastatin and glimepiride + rosuvastatin solutions characterized by peak concentration in plasma (*C*_max_), concentration peak time (*t*_max_), area under the concentration-time curve (AUC_(0–t)_, AUC_(0-∞)_), elimination rate constant (*K*_el_), clearance (Cl), and volume of distribution (*V*_d_) were derived by WinNonlin software version *5.0.1* (Pharsight Corporation, USA), using non compartmental analysis. Results are expressed as mean ± SEM. All pharmacokinetic parameters (except for *t*_max_) were logarithmically transformed before analysis. The pharmacokinetic variables were compared with a paired t-test (two-tailed) or, in the case of *t*_max_, by the Wilcoxon signed rank test. The level of statistical significance was P < 0.05. The calculated pharmacokinetic parameters were used for assessment of the drug interaction of glimepiride in coadministration with atorvastatin and rosuvastatin.

## RESULTS

### *In vitro* drug interaction study

Results of *in vitro* drug interaction study are shown in Table 1. Percentage metabolism of glimepiride alone was 9.06%. Percentage metabolism of glimepiride in glimepride + atorvastatin and glimepiride + rosuvastatin was 2.05% and 6.03%, respectively. Percentage metabolism of atorvastatin alone was 3.51%, whereas with coadministration of glimepiride it was 2.09%. Percentage metabolism of rosuvastatin alone was 0.47%, whereas coadministration with glimepiride showed 3.56% percentage metabolism.

**Table 1:** *In vitro* drug interaction studies of glimepride– atorvastatin and glimepride–rosuvastatin

Drug	Percentage metabolism
Glimepiride	9.06
Glimepiride − glimepiride + atorvastatin	2.05
Glimepiride − glimepiride + rosuvastatin	6.03
Atorvastatin	3.51
Atorvastatin − glimepiride + atorvastatin	2.09
Rosuvastatin	0.47
Rosuvastatin − glimepiride + rosuvastatin	3.56

### *In vivo* drug interaction study

**Figure 1:**
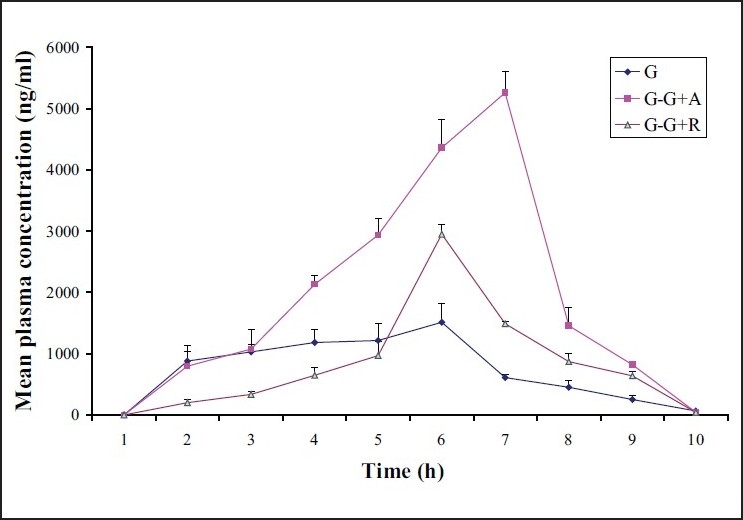
The mean plasma concentration–time curve for glimepride (G), glimepride coadministered with atorvastatin (G−G+A) and glimepride coadministered with rosuvastatin (G−G+R) in rats

The mean plasma concentration-time curve for glimepride (6 mg/kg, p.o.), glimepride coadministered with atorvastatin (60 mg/kg, p.o.) and glimepride coadministered with rosuvastatin (60 mg/kg, p.o.) is shown in Figure 1. Values of all pharmacokinetic parameters of *in vivo* study are shown in Table 2. The mean value of *t*_max_ for glimepiride alone was found to be 1.22 h; when glimepiride was coadministered with atorvastatin, *t*_max_ was found to be 3.33 h which was almost a three fold increase, while in the case of glimepiride coadministration with rosuvastatin *t*_max_ was found to be 2.00 h which was not a significant change when compared to glimepiride alone. The mean value of *C*_max_ (ng/ml) for glimepiride alone was found to be 1705.02; coadministration of glimepiride with atorvastatin resulted in a *C*_max_ 5318.27 which was almost a threefold increase, while in the case of glimepiride coadministration with rosuvastatin *C*_max_ was found to be 2950.20 which was not significant when compared to glimepiride alone. The mean value of AUC_(0—t)_ (h ng/ml) for glimepiride alone was found to be 8814.83; when glimepiride was coadministered with atorvastatin, AUC_(0-*t*)_ was found to be 30723.20, which was almost a fourfold increase while in the case of glimepiride coadministration with rosuvastatin, AUC_(0-*t*)_ was found to be 14812.67 which was not a significant change when compared to glimepiride alone. The mean value of AUC_(0-∞)_ (h ng/ml) for glimepiride alone was found to be 9489.44; glimepiride when coadministered with atorvastatin resulted in an AUC_(0-∞)_ of 30932.78 which was almost a threefold increase while in the case of glimepiride coadministration with rosuvastatin, AUC_(0-∞)_ was found to be 15717.01 which was not a significant change when compared to glimepiride alone. The mean value of *V*_d_ for glimepiride alone was found to be 6100.51 ml. When glimepiride was coadministered with atorvastatin, *V*_d_ was found to be 1011.81 ml and glimepiride coadministration with rosuvastatin resulted in a *V*_d_ of 1829.39 ml which was a significant decrease with both statins but more significant with atorvastatin. The mean value of Cl for glimepiride alone was found to be 666.03 ml/h, glimepiride when coadministered with atorvastatin gave a Cl of found to be 194.80 ml/h which was almost a threefold decrease, while in the case of glimepiride coadministration with rosuvastatin Cl was found to be 386.83 ml/h which was not that significant when compared to glimepiride alone. The mean value of *K*_el_ for glimepiride alone was found to be 0.11 h^-1^, glimepiride when coadministered with atorvastatin showed a *K*_el_ of 0.19 hr^-1^ and glimepiride coadministration with rosuvastatin resulted in a *K*_el_ of 0.22 h^-1^ which was almost a onefold increase with both the statins.

**Table 2:** Pharmacokinetic parameters of glimepride on coadministration with atorvastatin and rosuvastatin

Parameters	Glimepiride (6 mg/kg, p.o.)	Atorvastatin (60 mg/kg, p.o)	Rosuvastatin (60 mg/kg, p.o.)	Glimepiride (6 mg/kg, p.o.) + atorvastatin (60 mg/kg, p.o)	Glimepiride (6 mg/kg, p.o.) + rosuvastatin (60 mg/kg, p.o.)
*t*_max_ (h)	1.22 ± 0.4	2.00 ± 0.0	4.00 ± 2.0	3.33 ± 0.7*	2.00 ± 0.0
*C*_max_ (ng/ml)	1,705.02 ± 262.3	5,551.95 ± 549.4	2,079.19 ± 1,512.5	5,318.27 ± 291.1*	2,950.20 ± 159.57*
*T*_1/2_ (h)	6.53 ± 0.96	0.64 ± 0.02	1.65 ± 0.23	3.59 ± 0.14*	3.25 ± 0.30*
K_el_ (h^-1^)	0.11 ± 0.02	1.09 ± 0.04	0.43 ± 0.06	0.19 ± 0.01*	0.22 ± 0.02*
AUC_(0−t)_ (h ng/ml)	8,814.83 ± 1,335.4	12,130.48 ± 1,689.5	4,469.57 ± 2,887.3	30,723.20 ± 1,416.4*	14,200.25 ± 1,915.7
AUC_(0.−∞)_ (h ng/ml)	9,489.44 ± 1,618.5	12,144.64 ± 1,687.9	6,706.60 ± 3,038.9	30,932.78 ± 1,399.8*	15,717.01 ± 1,298.4
*V*_d_ (ml)	6,100.51 ± 896.3	4,731.69 ± 688.5	33,655.08 ± 18,262.2	1,011.81 ± 88.9*	1,829.39 ± 262.3*
Cl (ml/h)	666.03 ± 99.3	5,116.93 ± 636.4	12,928.17 ± 5,858.2	194.80 ± 9.22*	386.83 ± 30.79

## DISCUSSION

Drug interactions can lead to changed systemic exposure, resulting in variations in response of the coadministered drugs. The metabolic pathway of glimepiride is CYP2C9 and atorvastatin is CYP3A4. Glimepiride alone was showed a high metabolism but when coadministered with atorvastatin, atorvastatin inhibits its own metabolism as well as the metabolism of glimepiride. So, this shows that, atorvastatin acts as an enzyme inhibitor. Atorvastatin strongly inhibited CYP2C9-mediated glimepride metabolism *in vitro*, which reasonably agreed well with the observed four to fivefold increase of AUC in *in vivo* study. The metabolic pathway of both glimepiride and rosuvastatin is CYP2C9. Because of having the same metabolic pathway, when both the drugs are coadministered, rosuvastatin competes for CYP2C9 and inhibits the metabolism of glimepiride but not to the extent of atorvastatin. Besides, rosuvastatin induces its own metabolism too. So it shows that rosuvastatin acts as an enzyme inhibitor for glimepiride and is an enzyme inducer for its own.

The peak (*C*_max_) in the plasma concentration–time curve of glimepride + atorvastatin occurred at about 3.33 h, whereas with the glimepride alone it was seen at 1.22 h post administration, indicating that atorvastatin may have caused a delay in the rate of absorption of oral glimepride but enhanced the extent of absorption considering the significant difference between the AUC of glimepride alone and glimepride + atorvastatin group. There is no statistical difference in the peak plasma concentration of glimepride when it was coadministered with rosuvastatin. The elimination rate constant (*k*_el_) of a drug indicates the proportion of that drug that is removed from the body[15] and half-life is a reciprocal function of this. As administration of atorvastatin and rosuvastatin with glimepride increased the elimination rate constant of the glimepride especially in linear kinetic, they invariably caused a reduction in the half-life of glimepride. The liver is the main site of metabolism of glimepride and the whole drug is cleared from the systemic circulation by the liver.[8] Since atorvastatin and rosuvastatin decreased the clearance of the glimepride, it may be said that both statins altered the metabolism of the drug by the liver.

In conclusion, glimepride metabolism is little affected by rosuvastatin *in vitro*, and consequently, it predicted no drug—drug interaction between glimepride and rosuvastatin in humans, which agreed with the negligible interaction in *in vivo* study. On the other hand, atorvastatin could cause a decrease in the metabolism of glimepride *in vitro* and increase in the bioavailability of glimepride per oral but glimepride had no significant effect on the metabolism of atorvastatin when the two are dosed concomitantly. This may pose a positive implication in clinical practice.

Although further investigation of appropriate doses and dose regimens is warranted, the results of this study and accompanying simulations support the possibility of a reduction in both the magnitude and frequency of glimepride dosing when given in combination with atorvastatin but from safety point of view rosuvastatin is better to prescribe as a coadministration therapy with glimepiride.

## References

[1] Krall LP, Beaser RS (1996). Joslin Diabetes Manual.

[2] O’Brien RC, Luo M, Balazs N, Mercuri J (2000). *In vitro* and *in vivo* antioxidant properties of Gliclazide. J Diabetes Complications.

[3] Taskinen MR (1998). Strategies for the management of diabetic dyslipidemia. Drugs.

[4] Vijan S, Hayward RA; American College of Physicians (2004). Pharmacologic lipid-lowering therapy in type 2 diabetes mellitus: background paper for the American College of Physicians. Ann Intern Med.

[5] Snow V, Aronson MD, Hornbake ER, Mottur-Pilson C (2004). Clinical Efficacy Assessment Subcommittee of the American College of Physicians. Lipid control in the management of type 2 diabetes mellitus: a clinical practice guideline from the American College of Physicians. Ann Intern Med.

[6] Knopp RH, d’Emden M, Smilde JG, Pocock SJ (2006). Efficacy and safety of atorvastatin in the prevention of cardiovascular end points in subjects with type 2 diabetes. Diabetes Care.

[7] Adsule SM, Baig MS, Gade PR, Khandelwal PN (2009). A comparative evaluation of safety and efficacy of rosuvastatin, simvastatin, and atorvastatin in patients of type 2 diabetes mellitus with dyslipidemia. Int J Diabetes Dev Ctries.

[8] Langtry HD, Balfour JA (1998). Glimepiride: a review of its use in the management of type 2 diabetes mellitus. Drugs.

[9] Malinowski JM (1998). Atorvastatin: a hydroxymethylglutaryl-coenzyme A reductase inhibitor. *Am J Health Syst Pharm*.

[10] White CM (2002). A review of the pharmacologic and pharmacokinetic aspects of rosuvastatin. J Clin Pharmacol.

[11] Igel M, Sudhop T, Von-Bergmann K (2001). Metabolism and drug interaction of 3-hydroxy-3-methylglutaryl-coenzyme A reductase inhibitors (statins). Eur J Clin Pharmacol.

[12] Lin JH (2000). Sense and nonsense in the prediction of drug-drug interactions. Curr Drug Metab.

[13] Zhang L, Zhang YD, Zhao P, Huang SM (2009). Predicting drug-drug interactions: An FDA Perspective. AAPS J.

[14] Boxenbaum H, DiLea C (1995). First time in human dose selection: allometric thoughts and perspectives. J Clin Pharmacol.

[15] Proudfoot SG, Aulton ME (1999). Pharmaceutics: The Science of Dosage form design. Assessment of bioavailability.

